# *N* ^2^,*N* ^2′^-Bis(pyridin-2-yl­methyl­idene)pyridine-2,6-dicarbohydrazide dimethyl­formamide monosolvate

**DOI:** 10.1107/S1600536810039346

**Published:** 2010-10-09

**Authors:** Chuan-Gang Fan, Ming-zhi Song

**Affiliations:** a College of Chemistry and Chemical Technology, Binzhou University, Binzhou 256600, Shandong, People’s Republic of China

## Abstract

In the crystal of the title compound, C_22_H_22_N_8_O_3_, the dicarbohydrazide mol­ecules are linked into a chain along [010] by C—H⋯N inter­actions involving the pyridyl N atoms and aromatic C—H groups. The DMF mol­ecule is hydrogen bonded with the amide N—H *via* N—H⋯O inter­actions. C—H⋯O inter­actions also occur.

## Related literature

For the biological properties of Schiff base ligands, see: Bedia *et al.* (2006 ▶). For related structures, see: Alhadi *et al.* (2008 ▶); Nie (2008 ▶).
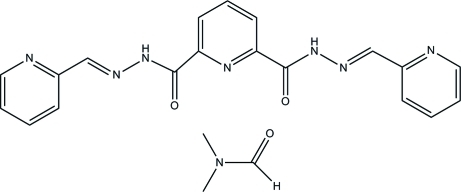


## Experimental

### 

#### Crystal data

C_22_H_22_N_8_O_3_
*M* *_r_* = 446.48
Monoclinic, 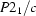

*a* = 10.0944 (9) Å
*b* = 24.639 (2) Å
*c* = 9.6552 (8) Å
β = 110.826 (2)°
*V* = 2244.5 (3) Å^3^
*Z* = 4
Mo *K*α radiation
μ = 0.09 mm^−1^
*T* = 298 K
0.36 × 0.31 × 0.17 mm

#### Data collection

Siemens SMART CCD area-detector diffractometer
Absorption correction: multi-scan (*SADABS*; Sheldrick, 1996 ▶) *T*_min_ = 0.967, *T*_max_ = 0.984
11220 measured reflections
3945 independent reflections
2291 reflections with *I* > 2σ(*I*)
*R*_int_ = 0.040

#### Refinement

*R*[*F* ^2^ > 2σ(*F* ^2^)] = 0.045
*wR*(*F* ^2^) = 0.126
*S* = 1.03
3945 reflections
300 parameters
H-atom parameters constrained
Δρ_max_ = 0.17 e Å^−3^
Δρ_min_ = −0.17 e Å^−3^

### 

Data collection: *SMART* (Siemens, 2007 ▶); cell refinement: *SAINT* (Siemens, 2007 ▶); data reduction: *SAINT*; program(s) used to solve structure: *SHELXS97* (Sheldrick, 2008 ▶); program(s) used to refine structure: *SHELXL97* (Sheldrick, 2008 ▶); molecular graphics: *SHELXTL* (Sheldrick, 2008 ▶); software used to prepare material for publication: *SHELXTL*.

## Supplementary Material

Crystal structure: contains datablocks I, global. DOI: 10.1107/S1600536810039346/ds2055sup1.cif

Structure factors: contains datablocks I. DOI: 10.1107/S1600536810039346/ds2055Isup2.hkl

Additional supplementary materials:  crystallographic information; 3D view; checkCIF report

## Figures and Tables

**Table 1:** Hydrogen-bond geometry (Å, °)

*D*—H⋯*A*	*D*—H	H⋯*A*	*D*⋯*A*	*D*—H⋯*A*
N2—H2⋯O3	0.86	2.29	3.015 (3)	142
N5—H5⋯O3	0.86	2.26	3.045 (3)	152
N2—H2⋯O3	0.86	2.29	3.015 (3)	142
N5—H5⋯O3	0.86	2.26	3.045 (3)	152
C22—H22*A*⋯O2^i^	0.96	2.59	3.469 (4)	152
C12—H12⋯O1^ii^	0.93	2.68	3.534 (3)	153
C11—H11⋯N4^iii^	0.93	2.62	3.402 (4)	142
C3—H3⋯N4^iv^	0.93	2.68	3.585 (3)	165
C5—H5*A*⋯N7^iv^	0.93	2.64	3.520 (4)	158

Symmetry codes: (i) 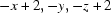; (ii) 
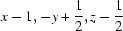
; (iii) 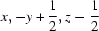; (iv) 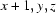.

**Fig. 1.**
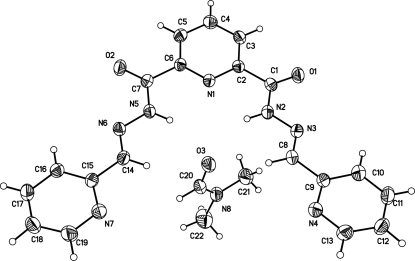
The content of asymmetric unit of the title compound showing the atomic numbering scheme and 30% probability displacement ellipsoids.

**Fig. 2.**
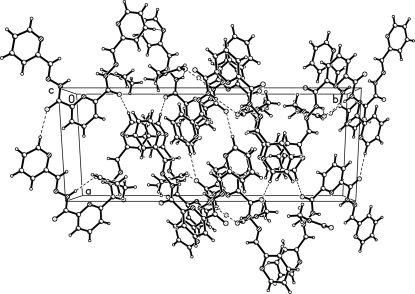
The crystal packing of (I), viewed along the a axis.

## supplementary crystallographic information

### Comment

Schiff bases containing pyridine ring have received considerable attention during the last decades, mainly because their steric and electronic properties can be easily adapted by choosing the right amine and aldehyde precursors(Bedia *et al.*, 2006).

We report here the crystal structure of the title new Schiff base compound (I).

In (I) (Fig. 1), the bond lengths and angles are normal and are comparable to the values observed in similar compounds (Nie *et al.*, 2008; Alhadi *et al.*, 2008). Meanwhile, the structure unit of (I), contains one *N*'^2^,*N*'^6^-bis(pyridin-2-ylmethylene)pyridine-2,6-dicarbohydrazide molecule and one *N*,*N*-dimethylformamide solvate molecule. In molecule *N*'^2^,*N*'^6^-bis (pyridin-2-ylmethylene)pyridine-2,6-dicarbohydrazide, the centre pyridine ring and the pyridine rings (n4/c9/*c*10/*c*11/*c*12/*c*13),(n1/c2/c3/c4/c5/c6) form the dihedral angles of 9.13 (15)°, 4.35 (17)°, respectively, which mean the atoms of the molecule *N*'^2^,*N*'^6^-bis (pyridin-2-ylmethylene)pyridine-2,6-dicarbohydrazide are almost coplanar.

Moreover, the crystal surpermolecular structure was built from the connections of weak intermolecular N—H···O, C—H···O and C—H···N as shown in table 1.

### Experimental

Pyridine-2,6-dicarbohydrazide (6 mmol) in DMF (20 ml) was added to pyridine-2-aldehyde (12 mmol). The mixture was refluxed with stirring for 6 h. A red precipitate was then obtained. Red crystals suitable for X-ray diffraction analysis formed after one week on slow evaporation of DMF solution at room temperature. Elemental analysis: calculated for C_22_H_22_N_8_O_3_: C 59.18, H 4.97, N 25.10%; found: C 59.28, H 4.82, N 25.21%.

### Refinement

All H atoms were placed in geometrically idealized positions (N—H 0.86 and C—H = 0.93–0.96 Å) and treated as riding on their parent atoms, with *U*_iso_(H) = 1.2U-1.5_eq_(C) (C,*N*).

### Crystal data

**Table:**

C_19_H_15_N_7_O_2_·C_3_H_7_NO	*F*(000) = 936
*M**_r_* = 446.48	*D*_x_ = 1.321 Mg m^−^^3^
Monoclinic, *P*2_1_/*c*	Mo *K*α radiation, λ = 0.71073 Å
Hall symbol: -P 2ybc	Cell parameters from 2269 reflections
*a* = 10.0944 (9) Å	θ = 2.3–22.5°
*b* = 24.639 (2) Å	µ = 0.09 mm^−^^1^
*c* = 9.6552 (8) Å	*T* = 298 K
β = 110.826 (2)°	Block, red
*V* = 2244.5 (3) Å^3^	0.36 × 0.31 × 0.17 mm
*Z* = 4	

### Data collection

**Table:**

Siemens SMART CCD area-detector diffractometer	3945 independent reflections
Radiation source: fine-focus sealed tube	2291 reflections with *I* > 2σ(*I*)
graphite	*R*_int_ = 0.040
phi and ω scans	θ_max_ = 25.0°, θ_min_ = 1.7°
Absorption correction: multi-scan (*SADABS*; Sheldrick, 1996)	*h* = −11→12
*T*_min_ = 0.967, *T*_max_ = 0.984	*k* = −25→29
11220 measured reflections	*l* = −11→9

### Refinement

**Table:**

Refinement on *F*^2^	Primary atom site location: structure-invariant direct methods
Least-squares matrix: full	Secondary atom site location: difference Fourier map
*R*[*F*^2^ > 2σ(*F*^2^)] = 0.045	Hydrogen site location: inferred from neighbouring sites
*wR*(*F*^2^) = 0.126	H-atom parameters constrained
*S* = 1.03	*w* = 1/[σ^2^(*F*_o_^2^) + (0.0422*P*)^2^ + 0.8005*P*] where *P* = (*F*_o_^2^ + 2*F*_c_^2^)/3
3945 reflections	(Δ/σ)_max_ < 0.001
300 parameters	Δρ_max_ = 0.17 e Å^−^^3^
0 restraints	Δρ_min_ = −0.17 e Å^−^^3^

### Special details

**Table:**

Geometry. All e.s.d.'s (except the e.s.d. in the dihedral angle between two l.s. planes) are estimated using the full covariance matrix. The cell e.s.d.'s are taken into account individually in the estimation of e.s.d.'s in distances, angles and torsion angles; correlations between e.s.d.'s in cell parameters are only used when they are defined by crystal symmetry. An approximate (isotropic) treatment of cell e.s.d.'s is used for estimating e.s.d.'s involving l.s. planes.
Refinement. Refinement of *F*^2^ against ALL reflections. The weighted *R*-factor *wR* and goodness of fit *S* are based on *F*^2^, conventional *R*-factors *R* are based on *F*, with *F* set to zero for negative *F*^2^. The threshold expression of *F*^2^ > σ(*F*^2^) is used only for calculating *R*-factors(gt) *etc*. and is not relevant to the choice of reflections for refinement. *R*-factors based on *F*^2^ are statistically about twice as large as those based on *F*, and *R*- factors based on ALL data will be even larger.

### Fractional atomic coordinates and isotropic or equivalent isotropic displacement parameters (Å^2^)

**Table:**

	*x*	*y*	*z*	*U*_iso_*/*U*_eq_	
N1	1.0532 (2)	0.06010 (8)	0.6046 (2)	0.0399 (5)	
N2	0.8597 (2)	0.13178 (8)	0.4467 (2)	0.0478 (6)	
H2	0.8437	0.1064	0.5000	0.057*	
N3	0.7529 (2)	0.16653 (8)	0.3698 (2)	0.0468 (6)	
N4	0.3891 (2)	0.17800 (9)	0.3134 (3)	0.0544 (6)	
N5	0.9686 (2)	−0.00721 (8)	0.7745 (2)	0.0453 (5)	
H5	0.9187	0.0193	0.7246	0.054*	
N6	0.9148 (2)	−0.04071 (8)	0.8553 (2)	0.0446 (5)	
N7	0.5832 (2)	−0.05299 (9)	0.8981 (3)	0.0593 (7)	
N8	0.8324 (2)	0.16209 (9)	0.8702 (2)	0.0538 (6)	
O1	1.02185 (19)	0.17371 (7)	0.3714 (2)	0.0590 (5)	
O2	1.1763 (2)	−0.05273 (8)	0.8419 (2)	0.0643 (6)	
O3	0.7810 (2)	0.09334 (7)	0.7038 (2)	0.0647 (6)	
C1	0.9891 (3)	0.13729 (10)	0.4390 (3)	0.0435 (6)	
C2	1.0946 (3)	0.09502 (9)	0.5217 (3)	0.0397 (6)	
C3	1.2263 (3)	0.09326 (10)	0.5094 (3)	0.0483 (7)	
H3	1.2512	0.1182	0.4506	0.058*	
C4	1.3202 (3)	0.05384 (11)	0.5861 (3)	0.0564 (8)	
H4	1.4097	0.0516	0.5794	0.068*	
C5	1.2802 (3)	0.01771 (11)	0.6728 (3)	0.0524 (7)	
H5A	1.3422	−0.0092	0.7256	0.063*	
C6	1.1465 (3)	0.02221 (9)	0.6800 (3)	0.0405 (6)	
C7	1.1003 (3)	−0.01612 (10)	0.7739 (3)	0.0431 (6)	
C8	0.6315 (3)	0.15664 (10)	0.3761 (3)	0.0489 (7)	
H8	0.6194	0.1272	0.4308	0.059*	
C9	0.5108 (3)	0.19145 (10)	0.2974 (3)	0.0454 (7)	
C10	0.5215 (3)	0.23516 (11)	0.2119 (3)	0.0607 (8)	
H10	0.6081	0.2438	0.2036	0.073*	
C11	0.4044 (4)	0.26539 (12)	0.1401 (3)	0.0659 (9)	
H11	0.4100	0.2950	0.0828	0.079*	
C12	0.2779 (3)	0.25150 (12)	0.1535 (3)	0.0621 (8)	
H12	0.1958	0.2711	0.1044	0.075*	
C13	0.2757 (3)	0.20829 (12)	0.2407 (3)	0.0614 (8)	
H13	0.1899	0.1993	0.2502	0.074*	
C14	0.7866 (3)	−0.03185 (10)	0.8425 (3)	0.0466 (7)	
H14	0.7361	−0.0040	0.7815	0.056*	
C15	0.7184 (3)	−0.06509 (10)	0.9230 (3)	0.0429 (6)	
C16	0.7895 (3)	−0.10600 (10)	1.0182 (3)	0.0495 (7)	
H16	0.8846	−0.1128	1.0347	0.059*	
C17	0.7184 (3)	−0.13655 (11)	1.0882 (3)	0.0573 (8)	
H17	0.7637	−0.1647	1.1513	0.069*	
C18	0.5798 (3)	−0.12467 (12)	1.0632 (3)	0.0619 (8)	
H18	0.5286	−0.1446	1.1089	0.074*	
C19	0.5177 (3)	−0.08291 (13)	0.9697 (4)	0.0680 (9)	
H19	0.4237	−0.0748	0.9550	0.082*	
C20	0.7886 (3)	0.11313 (12)	0.8238 (3)	0.0567 (8)	
H20	0.7607	0.0912	0.8870	0.068*	
C21	0.8856 (4)	0.19753 (12)	0.7832 (4)	0.0753 (10)	
H21A	0.9833	0.1892	0.8016	0.113*	
H21B	0.8776	0.2346	0.8101	0.113*	
H21C	0.8314	0.1924	0.6799	0.113*	
C22	0.8389 (4)	0.18074 (14)	1.0149 (4)	0.0905 (12)	
H22A	0.8091	0.1521	1.0646	0.136*	
H22B	0.7774	0.2115	1.0035	0.136*	
H22C	0.9344	0.1910	1.0724	0.136*	

### Atomic displacement parameters (Å^2^)

**Table:**

	*U*^11^	*U*^22^	*U*^33^	*U*^12^	*U*^13^	*U*^23^
N1	0.0422 (12)	0.0380 (11)	0.0372 (12)	−0.0013 (10)	0.0111 (10)	−0.0001 (10)
N2	0.0479 (14)	0.0483 (13)	0.0485 (14)	0.0057 (11)	0.0187 (12)	0.0119 (11)
N3	0.0480 (14)	0.0495 (13)	0.0416 (14)	0.0077 (11)	0.0145 (11)	0.0060 (11)
N4	0.0495 (14)	0.0638 (15)	0.0505 (15)	0.0082 (12)	0.0186 (12)	0.0115 (12)
N5	0.0477 (13)	0.0416 (12)	0.0483 (14)	0.0007 (10)	0.0192 (11)	0.0087 (10)
N6	0.0494 (14)	0.0399 (12)	0.0470 (14)	−0.0032 (10)	0.0203 (12)	0.0024 (10)
N7	0.0485 (15)	0.0632 (15)	0.0695 (18)	0.0088 (12)	0.0250 (13)	0.0170 (13)
N8	0.0674 (16)	0.0472 (14)	0.0459 (15)	0.0036 (12)	0.0189 (13)	0.0027 (12)
O1	0.0569 (12)	0.0580 (12)	0.0641 (14)	0.0013 (9)	0.0240 (11)	0.0191 (10)
O2	0.0577 (13)	0.0570 (12)	0.0801 (16)	0.0126 (10)	0.0268 (12)	0.0261 (11)
O3	0.0962 (16)	0.0493 (11)	0.0570 (14)	0.0045 (11)	0.0377 (13)	0.0016 (10)
C1	0.0471 (16)	0.0466 (16)	0.0365 (16)	−0.0029 (13)	0.0144 (13)	0.0008 (13)
C2	0.0436 (15)	0.0393 (14)	0.0345 (15)	−0.0028 (12)	0.0118 (12)	−0.0021 (12)
C3	0.0510 (17)	0.0504 (16)	0.0463 (17)	−0.0022 (13)	0.0207 (14)	0.0058 (13)
C4	0.0468 (17)	0.0632 (18)	0.064 (2)	0.0018 (15)	0.0250 (16)	0.0072 (16)
C5	0.0450 (16)	0.0546 (17)	0.0570 (19)	0.0076 (13)	0.0173 (15)	0.0065 (15)
C6	0.0443 (15)	0.0358 (14)	0.0386 (16)	−0.0006 (12)	0.0113 (13)	−0.0014 (12)
C7	0.0447 (16)	0.0377 (14)	0.0448 (17)	0.0009 (12)	0.0134 (14)	0.0009 (12)
C8	0.0548 (18)	0.0493 (16)	0.0440 (17)	0.0065 (14)	0.0194 (14)	0.0116 (13)
C9	0.0484 (16)	0.0496 (16)	0.0381 (16)	0.0049 (13)	0.0152 (14)	0.0029 (13)
C10	0.063 (2)	0.0606 (19)	0.062 (2)	0.0068 (16)	0.0265 (17)	0.0139 (16)
C11	0.081 (2)	0.0568 (19)	0.057 (2)	0.0143 (17)	0.0205 (18)	0.0143 (15)
C12	0.061 (2)	0.067 (2)	0.051 (2)	0.0235 (17)	0.0111 (16)	0.0045 (16)
C13	0.0514 (18)	0.075 (2)	0.058 (2)	0.0107 (16)	0.0188 (16)	0.0044 (17)
C14	0.0503 (17)	0.0375 (14)	0.0518 (18)	0.0025 (13)	0.0179 (14)	0.0038 (13)
C15	0.0437 (16)	0.0384 (14)	0.0444 (16)	−0.0019 (12)	0.0128 (13)	−0.0015 (12)
C16	0.0457 (16)	0.0472 (16)	0.0559 (18)	0.0020 (13)	0.0183 (14)	0.0030 (14)
C17	0.061 (2)	0.0515 (17)	0.057 (2)	0.0003 (15)	0.0191 (16)	0.0122 (15)
C18	0.059 (2)	0.0658 (19)	0.065 (2)	−0.0070 (16)	0.0279 (17)	0.0115 (17)
C19	0.0472 (18)	0.082 (2)	0.078 (2)	0.0073 (16)	0.0266 (17)	0.0209 (19)
C20	0.072 (2)	0.0517 (18)	0.053 (2)	0.0035 (15)	0.0305 (17)	0.0106 (15)
C21	0.094 (3)	0.0557 (19)	0.086 (3)	−0.0023 (17)	0.044 (2)	0.0063 (18)
C22	0.132 (3)	0.083 (2)	0.053 (2)	−0.004 (2)	0.030 (2)	−0.0114 (18)

### Geometric parameters (Å, °)

**Table:**

N1—C2	1.339 (3)	C6—C7	1.494 (3)
N1—C6	1.342 (3)	C8—C9	1.463 (3)
N2—C1	1.342 (3)	C8—H8	0.9300
N2—N3	1.370 (3)	C9—C10	1.384 (3)
N2—H2	0.8600	C10—C11	1.360 (4)
N3—C8	1.272 (3)	C10—H10	0.9300
N4—C9	1.333 (3)	C11—C12	1.372 (4)
N4—C13	1.338 (3)	C11—H11	0.9300
N5—C7	1.349 (3)	C12—C13	1.362 (4)
N5—N6	1.373 (3)	C12—H12	0.9300
N5—H5	0.8600	C13—H13	0.9300
N6—C14	1.275 (3)	C14—C15	1.459 (3)
N7—C15	1.332 (3)	C14—H14	0.9300
N7—C19	1.336 (3)	C15—C16	1.381 (3)
N8—C20	1.308 (3)	C16—C17	1.373 (3)
N8—C21	1.441 (3)	C16—H16	0.9300
N8—C22	1.450 (4)	C17—C18	1.364 (4)
O1—C1	1.222 (3)	C17—H17	0.9300
O2—C7	1.215 (3)	C18—C19	1.365 (4)
O3—C20	1.234 (3)	C18—H18	0.9300
C1—C2	1.500 (3)	C19—H19	0.9300
C2—C3	1.377 (3)	C20—H20	0.9300
C3—C4	1.375 (3)	C21—H21A	0.9600
C3—H3	0.9300	C21—H21B	0.9600
C4—C5	1.378 (4)	C21—H21C	0.9600
C4—H4	0.9300	C22—H22A	0.9600
C5—C6	1.380 (3)	C22—H22B	0.9600
C5—H5A	0.9300	C22—H22C	0.9600
			
C2—N1—C6	117.6 (2)	C9—C10—H10	120.3
C1—N2—N3	120.0 (2)	C10—C11—C12	119.0 (3)
C1—N2—H2	120.0	C10—C11—H11	120.5
N3—N2—H2	120.0	C12—C11—H11	120.5
C8—N3—N2	116.1 (2)	C13—C12—C11	118.3 (3)
C9—N4—C13	116.9 (2)	C13—C12—H12	120.9
C7—N5—N6	119.6 (2)	C11—C12—H12	120.9
C7—N5—H5	120.2	N4—C13—C12	124.2 (3)
N6—N5—H5	120.2	N4—C13—H13	117.9
C14—N6—N5	115.8 (2)	C12—C13—H13	117.9
C15—N7—C19	116.5 (2)	N6—C14—C15	120.5 (2)
C20—N8—C21	120.6 (2)	N6—C14—H14	119.8
C20—N8—C22	121.1 (3)	C15—C14—H14	119.8
C21—N8—C22	118.1 (3)	N7—C15—C16	122.7 (2)
O1—C1—N2	123.9 (2)	N7—C15—C14	115.1 (2)
O1—C1—C2	121.2 (2)	C16—C15—C14	122.2 (2)
N2—C1—C2	114.9 (2)	C17—C16—C15	119.2 (2)
N1—C2—C3	123.1 (2)	C17—C16—H16	120.4
N1—C2—C1	116.8 (2)	C15—C16—H16	120.4
C3—C2—C1	120.0 (2)	C18—C17—C16	118.6 (3)
C4—C3—C2	118.6 (2)	C18—C17—H17	120.7
C4—C3—H3	120.7	C16—C17—H17	120.7
C2—C3—H3	120.7	C17—C18—C19	118.7 (3)
C3—C4—C5	119.2 (2)	C17—C18—H18	120.7
C3—C4—H4	120.4	C19—C18—H18	120.7
C5—C4—H4	120.4	N7—C19—C18	124.3 (3)
C4—C5—C6	118.8 (3)	N7—C19—H19	117.9
C4—C5—H5A	120.6	C18—C19—H19	117.9
C6—C5—H5A	120.6	O3—C20—N8	125.9 (3)
N1—C6—C5	122.6 (2)	O3—C20—H20	117.0
N1—C6—C7	117.3 (2)	N8—C20—H20	117.0
C5—C6—C7	120.1 (2)	N8—C21—H21A	109.5
O2—C7—N5	123.5 (2)	N8—C21—H21B	109.5
O2—C7—C6	121.6 (2)	H21A—C21—H21B	109.5
N5—C7—C6	114.9 (2)	N8—C21—H21C	109.5
N3—C8—C9	120.1 (2)	H21A—C21—H21C	109.5
N3—C8—H8	119.9	H21B—C21—H21C	109.5
C9—C8—H8	119.9	N8—C22—H22A	109.5
N4—C9—C10	122.2 (2)	N8—C22—H22B	109.5
N4—C9—C8	115.2 (2)	H22A—C22—H22B	109.5
C10—C9—C8	122.6 (2)	N8—C22—H22C	109.5
C11—C10—C9	119.5 (3)	H22A—C22—H22C	109.5
C11—C10—H10	120.3	H22B—C22—H22C	109.5

### Hydrogen-bond geometry (Å, °)

**Table:**

*D*—H···*A*	*D*—H	H···*A*	*D*···*A*	*D*—H···*A*
N2—H2···O3	0.86	2.29	3.015 (3)	142
N5—H5···O3	0.86	2.26	3.045 (3)	152
N2—H2···O3	0.86	2.29	3.015 (3)	142
N5—H5···O3	0.86	2.26	3.045 (3)	152
C22—H22A···O2^i^	0.96	2.59	3.469 (4)	152
C12—H12···O1^ii^	0.93	2.68	3.534 (3)	153
C11—H11···N4^iii^	0.93	2.62	3.402 (4)	142
C3—H3···N4^iv^	0.93	2.68	3.585 (3)	165
C5—H5A···N7^iv^	0.93	2.64	3.520 (4)	158

Symmetry codes: (i) −*x*+2, −*y*, −*z*+2; (ii) *x*−1, −*y*+1/2, *z*−1/2; (iii) *x*, −*y*+1/2, *z*−1/2; (iv) *x*+1, *y*, *z*.
